# The Skin

**Published:** 1870-07

**Authors:** 


### THE SKIN.

BY ISHMAEL.

This is what we wear perpetually over our bones and next to our under-garments.

As every one knows, it is the outer covering of the body, a sort of a protection that stretches over the whole frame, tough, pliable and strong, holding in their places, as the silk of an umbrella holds its bones and ribs, the bones, muscles and sinews of the human body. But every one must not suppose that because he has heard of people who are said to be "thick-skinned" or "thin-skinned," that they are in reality so, although indeed, there are different kinds of skin af different thicknesses.

All animals and vegetables have what we may call a skin, and possesses it from the instant of their formation. It covers equally the seed and the egg. Any seed, if examined, will show a thin, but stout and almost impervious coating, and any one can see, between the shell and the meat of a boiled egg, if they choose to look, a thin sort of a membrane that tears like wet paper, which is the skin.

Possessing a nature far less corruptible than that which it encloses, the skin is able to preserve egg or seed for a long time from decay or destruction.

In some instances, this power of preservation is exerted in a lamentable manner. It protects the eggs of the gnat, the seventeen year locuss', and the palmer worm, which appears once only in thirty years, until a good warm place is found for them in which to hatch, and then it lets go its hold and the animal comes forth. It protects, too, the caterpillar in its chrysalid state, enabling it after many weeks to be transformed into a butterfly.

A curious instance of its power is shown in the egg of the horse gad-fly, that little winged devil which is such a pest to horses. Hard to believe as it may be, it is nevertheless a fact that this fly can never be hatched except inside the stomach of a horse! The average horse is not such a fool as to open its mouth and let a Mrs. Gadfly go down into its stomach for purposes of maternity, and the query would naturally follow: How does the egg get to its appointed place? It *must* go there somehow, for every summer sees the birth of many a gadfly.

This is the *modus operandi* :*

The eggs are deposited on the hairs of the horse, stuck there by a viscous matter with which the maternal gadfly is plentifully provided; the itching they excite upon the horse's skin compels him to lick the irritated part with his tongue, and by this simple method they are conveyed to the mouth and thus to the stomach, their thick skin protecting them from utter destruction.

Did you ask how they escaped from the stomach after they were hatched?

Ah! but that you must guess. I am concerned now, only with the integument which protects the egg until it arrives at its proper place. After it gets there, I have nothing more to do with it. You may rest assured, however, that as Nature has provided so admirable and simple a method for its entrance, its exit will be in an equally simple if not as admirable a manner.

To show still further the fact of which I am speaking, it is well known that eggs perfectly impregnated have been preserved untainted, by the incorruptibility of their skin for a long period of years. A French gentleman, by name BOMAGE, found three eggs once, that, protected from the air, he affirms must have lain in the wall of a church where they were discovered as fresh as though just laid, for a period of three hundred years. By what reasoning he arrived at such a conclusion, he does not inform us, and it is not necessary perhaps, as every one knows who BOMAGE is.

The writer of this is able to beat that "yarn." He saw an egg once, as fresh as though a Madame Hen had just triumphantly cackled over its production. And whatever feathered biped laid it, had a right to feel proud. It was three inches long and was double; that is, inside it was another perfectly formed egg, shell and all complete. The wonder of it was, however, that on the outer shell was very neatly painted the following names and date:

NOAH.
SHEM.
HAM.
JAPHET.
2348, B. C.

It will thus be seen what preservative virtue there is in the skin or outer integument that surrounds an egg. I cannot aver this, however, of all eggs, a statement that almost any groceryman will verify, although the skin is so impervious even in bad ones that, not until it is broken, is it at all evident to the outer air *how* bad they are.

*In a medical Journal of the dignity of the BISTOURY, it is well, now and then, to fling in a little Latin.

We have reason to be very thankful for the impervious nature and incorruptibility of this outer integument that surrounds seeds, for by it they are preserved from season to season and saved up for planting. Just peel off the filmy, almost imperceptible covering that surrounds a kernel of corn, a pumpkin seed or a kernel of wheat, save it up through the winter and plant it in the spring. You will discover how necessary its skin is to it or to its preservation for purposes of propagation.

This wise protection afforded by Nature, has largely been the means whereby mankind has been enabled to transfer the cereals and flowers of one country to another. It would have been a task almost, if not quite, impossible to transplant the tropical shrubs that bloom with such fragrance and such luxuriant foliage under the equatorial sun, to the colder climes; the tulip might have remained forever confined to Holland, Indian corn to America, potatoes to the tropics, turnips to Europe or tomatoes to Peru and Mexico, had it not been for this fine integument that surrounds and protects from decay the seeds of these various productions, this integument which, being of a similar nature to and provided for a similar purpose as that of our own bodies, we may call the skin.

Some other noticeable instances connected with this transfer of seeds from one land to another, and the springing up of plants and trees in countries unindigenous to them, without the aid of man, should not be passed over here.—Often on lonely islands in the sea where can be discovered nothing but bare, bleak rock, a tree will shoot up; on the highest peaks of the loftiest rocks or on the top of the highest steeples a little shrub will appear as if by magic, for certainly no human hand could or perhaps would plant there a seed.

It is still to the skin that these wee mites of seed owe their safety and ability to grow.—Some of them are provided with little hooks, and by means of them fasten themselves to the skins of animals and are carried about from place to place; others attach themselves by a native glue to the feathers of water-fowls and are washed off in distant seas, while a third sort are provided by nature with little downy wings, with which they rise in the atmosphere and are blown about by the breezes towards every quarter of the compass. Of the latter class are the seeds of a species of the birch tree which is often seen growing in those unaccessible spots just referred to.

Seeds also are preserved for a great length of time by their skin, some of them like the melon are improved the older they grow. A French gentleman sowed some seeds belonging to a collection, forty-five years after the collection had been made. There were three hundred and fifty distinct species. Nearly all of them proved productive. The seed business it will therefore be seen is a profitable calling; no wonder GRANT THORBURN got rich at it, and lived to be nearly one hundred years of age. One cannot very well get "stuck" on his stock. If not good for one season it will be good for the next.

I shall proceed in the next paper to spesk of the skin of animals and men, and its appendages. It is a subject replete with interest.

